# Role of region-of-interest magnetic resonance imaging fusion biopsy in mitigating overtreatment of localized prostate cancer – A retrospective cohort study

**DOI:** 10.1016/j.ejro.2025.100642

**Published:** 2025-03-07

**Authors:** Carrie Wang, Purvish Trivedi, Esther Katende, Varun Awasthi, Riley Smith, Ryan Putney, Yahya Bondokji, Jong Y. Park, Jasreman Dhillon, Kosj Yamoah

**Affiliations:** a Dartmouth Hitchcock Medical Center, Lebanon, NH, USA; b H. Lee Moffitt Cancer Center & Research Institute, Tampa, FL, USA; c Amity University, Noida, India

**Keywords:** Prostate cancer, Multiparametric magnetic resonance imaging, Active surveillance, Region of interest

## Abstract

**Background:**

Traditional ultrasonography-based prostate biopsy uses a transrectal approach for systematic sampling of 12 cores. The magnetic resonance imaging (MRI) fusion biopsy uses a targeted approach, first identifying regions of interest (ROI) clinically suspicious for prostate cancer (PCa) through MRI, before performing a prostate biopsy aided by ultrasonography.

**Methods:**

The single-center institutional retrospective cohort study used 442 men who were recommended for localized PCa management. Cohort A (n = 346) comprised patients who underwent MRI-guided TRUS biopsies, which included both standard 12-core TRUS biopsies and MRI-targeted biopsies performed simultaneously. Cohort B (n = 96) comprised patients who received only standard TRUS biopsy. The primary endpoint was Gleason reclassification, defined as the change in Gleason scores between standard TRUS and targeted region-of-interest (ROI) biopsies among cohort A. Secondary endpoint assessed the role of ROI biopsies in mitigating overtreatment by analyzing the probability of undergoing treatment and the duration of active surveillance (AS).

**Results:**

Among men classified as no tumor on standard biopsy, 16.9 % showed Gleason disease on subsequent ROI biopsy. Additionally, ROI group also had a longer time to receive primary treatment (*P* = .017), as they were more likely to opt for AS (54 %). Lastly, median time spent on AS was longer for the ROI group compared with the non-ROI cohort (*P* = .002).

**Conclusion:**

Adding multiparametric MRI (mpMRI) biopsy to standard TRUS biopsy may increase the detection of PCa. Additionally, mpMRI may allow patients to remain safely on AS, thereby reducing the need of prostate biopsies and improving cost-effectiveness.

## 1 Introduction

With an incidence rate of 111.3 per 100 000 men per year, prostate cancer (PCa) comprises a full 13.1 % of all new cancer cases per annum. [1] Many of these cases are diagnosed after a prostate biopsy. This approach is taken on the basis of an elevated prostate-specific antigen (PSA) level or an abnormal digital-rectal exam; thus, the patients undergoing this procedure are at an elevated risk for prostate malignancy. [2] Because of this, identifying possible tumors accurately and efficiently is paramount. According to SEER data, 5-year survival rate of localized PCa is almost 100 %. If PCa is not diagnosed until distant metastasis, the survival rate plummets to 30 %. [1] Prostate cancer screenings therefore represent a clinically crucial procedure to reduce PCA-specific mortality.

Thus, clarifying the utility of MRI-guided prostate biopsies as a screening tool is a pressing need. Traditional ultrasonography-based prostate biopsy uses a transrectal approach to obtain a random sampling of 12 cores, with 2 in each sextant of the prostate. This blind sampling is necessitated by the inability of ultrasound to visualize distinct regions of interest (ROI). In contrast, the MRI fusion biopsy uses a targeted approach, first identifying ROI that are clinically suspicious for PCa by using MRI, before performing a prostate biopsy aided by ultrasonography to target the previously identified ROI. [3] Existing research has suggested that targeted MRI fusion biopsies are a superior method of detecting PCa [4] and may offer a higher rate of detection of clinically significant PCa (csPCa), [5], [6] while simultaneously decreasing detection of clinically insignificant PCa [7] when used in conjunction with a standard biopsy. Furthermore, MRI fusion biopsy offers higher sensitivity than TRUS biopsy alone, ranging from approximately 87–93 %, depending on the criteria used to define csPCa [8].

The incorporation of novel biomarkers and liquid biopsy techniques is transforming patient selection and personalized cancer diagnosis [6]. Integrating advanced imaging with molecular diagnostics offers potential for enhancing active surveillance strategies and reducing overtreatment [9]. Despite increasing evidence of the utility of MRI biopsy, definitive guidelines on appropriate usage of it in clinically managing PCa have not been fully developed yet.

Early and accurate diagnosis is essential for optimizing clinical outcomes while reducing the risk of overtreatment [10]. Therefore, the primary objective of the study was to determine the effectiveness of MRI biopsy in improving the detection rate of clinically significant PCa. The secondary objective evaluated the role of MRI biopsy in mitigating overtreatment by examining the time-to-treatment from the first diagnosis, as well as in long-term management of clinically insignificant PCa treated with active surveillance (AS).

## 2 Methods

### 2.1 Study design and population

**Fig. 1:**
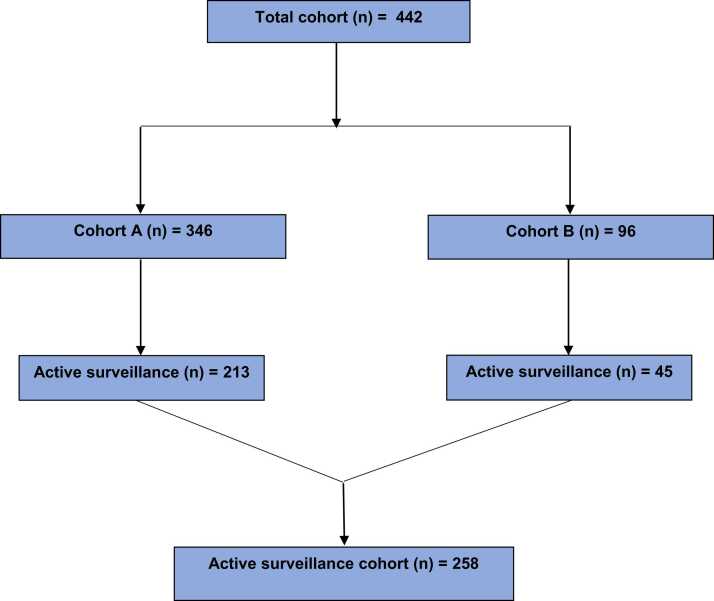
Cohort diagram. The study comprised of 442 patients. Out of which, 346 patients belonged to cohort A, whereas cohort B comprised of 96 patients. A total of 258 patients undergone active surveillance.

This single-center institutional retrospective cohort study used 442 men who were recommended for localized PCa management (Fig. 1). Patients were screened between 2011 and 2018. Cohort A (n = 346) and cohort B (n = 96) were identified based on whether or not they underwent an mpMRI study (Fig. 1). Cohort A consisted of patients who underwent MRI-guided TRUS biopsy, whereas cohort B consisted of patients who received only standard TRUS biopsy. Among patients in cohort A, both standard 12-core TRUS biopsy and MRI-guided ROI targeted biopsies were performed within the same session. There were no specific exclusion criteria for the study, however patients were excluded if they had received any prior treatment related to PCa; or had any contraindications to MRI such as claustrophobia, pacemaker, and medical implants. The initial focus among cohort A was to elucidate the role of ROI MRI fusion biopsy in detecting clinically significant PCa. We followed STROBE guidelines. [11] This study was approved by the institutional review board. Analyses were conducted with an approved waiver for obtaining informed consent and with authorization by the Health Insurance Portability and Accountability Act (HIPAA) of 1996. Patient information was deidentified and study protocol adhered to HIPAA guidelines regarding data privacy and security.

### 2.2 Participants follow-up

Among cohort A (n = 346), total duration of follow-up was calculated starting from the MRI-guided biopsy date to the subsequent treatment start date (date of radical prostatectomy or radiation start date). Similarly, among cohort B (n = 96), total duration of follow-up was calculated starting from the standard TRUS biopsy date to the subsequent treatment start date (date of radical prostatectomy or starting date of radiation therapy). When calculating the duration on AS, the day of the first biopsy was used as the starting point and was followed until the start date of subsequent treatment. For patients without treatment information, their last follow-up date was used to calculate total follow-up duration.

### 2.3 Outcome assessment

#### 2.3.1 Gleason reclassification

Gleason scores were categorized into 5 grade groups (GG1–5) according to the International Society of Urological Pathology consensus. Grade group 1 is considered clinically nonsignificant PCa with a corresponding Gleason score of equaled to 3 + 3. Grade groups 2 and 3 are considered clinically significant PCa, both with Gleason scores of 7 (group 2 = 3 +4) and (group 3 = 4 + 3). Grade groups 4 and 5 are also considered clinically significant PCa with Gleason scores of 8 (group 4) and 9–10 (group 5). [12] Region of interests were excluded from the initial 12- core biopsies. Therefore, the men in cohort A who received a diagnosis of *no tumor* and were classified as grade group 1 after standard TRUS biopsy, we compared these findings with their subsequent ROI biopsy findings. If the subsequent ROI biopsy produced a different Gleason grade group than the original standard TRUS biopsy, these patients were considered to have undergone a Gleason reclassification (Supplementary table 1). We intended to analyze whether or not a Gleason reclassification would have an impact on treatment decision-making, which we would indirectly track as a change in time-to-treatment.

#### 2.3.2 Time-to-treatment analysis

We wanted to investigate if the addition of an ROI biopsy would affect a patient’s likelihood of receiving treatment and/or their duration of AS. A total of 258 patients from both cohorts underwent AS (Fig. 1). The median duration patients remained on AS was compared between both cohort A and B (Fig. 1). Additionally, We aimed to assess the effectiveness of targeted ROI biopsies in identifying increase in Gleason grade group among patients undergoing AS. Therefore, within cohort A (n = 213) of patients who had AS, we concentrated on those diagnosed with no tumor and classified as grade group 1 following the standard TRUS biopsy. If the subsequent ROI biopsy revealed a higher Gleason grade group from the initial standard TRUS biopsy, these patients were regarded as having experienced Gleason reclassification. Among cohort A, We also compared changes in percent-positive biopsy cores (PPB) (n = 268) between ROI targeted biopsy and standard biopsy to evaluate PPB increase as a predictor of time-to-treatment. Lastly, we examined the relationship between different treatment types and ROI biopsy to determine if ROI biopsy is linked to delays in treatment.

#### 2.3.3 Statistical analysis

Demographic and clinicopathologic data were annotated by clinical-chart abstraction. Baseline characteristics were compared using a Chi-square test for categorical variables. Gleason reclassification was used as the main outcome among cohort A. For patients who underwent reclassification, logistic regression was performed to assess the association between reclassification and treatment decision-making. Potential confounders were identified based on clinical relevance and American Urological Association (AUA) guidelines for early detection of PCa and included (e.g., race, age at diagnosis, and prebiopsy PSA) [13], [14]. we used a multivariable logistic regression model adjusting for these confounders to estimate adjusted odds ratios and 95 % CI. Collinearity among covariates was assessed using variance inflation factors (VIF), and variables with VIF > 5 were excluded. Model fit was evaluated using the Hosmer-Lemeshow goodness-of-fit test. The impact of ROI biopsies on time-to-treatment and likelihood of receiving primary treatment were evaluated using a Kaplan–Meier curve with 95 % CI limits. The Cox proportional hazards model was used for time-dependent variables to report adjusted hazard ratio (aHR). We investigated whether ROI biopsy is correlated with increasing time-to-treatment. For patients who underwent AS, we also compared the duration spent on AS between the ROI and non-ROI groups using a T test. Comparison between treatment types (AS, prostatectomy, and radiation) and usage of ROI biopsy (yes vs no) was assessed to explain the association of ROI biopsy and primary intervention among localized PCa patients. Furthermore, another Kaplan–Meier curve was generated to compare how an increase in PPB after MRI fusion biopsy could have an impact on AS, thereby using PPB as a predictor of time-to-treatment for cohort A. A 2-sided alpha value of ≤ 0.05 was considered statistically significant. Analysis was carried out using SAS, Version 9.4 software.

## 3 Results

Baseline cohort characteristics and MRI findings are summarized in Table 1. The median age for patients who did not undergo ROI biopsy (69.5 years, IQR 65–74.5) was similar to the median age for patients who did undergo ROI biopsy (69 years, IQR 62–73). There was no statistically significant difference between both ROI categories in terms of age at diagnosis. However, there was a statistically significant difference (*P* = .03) between both ROI cohorts in terms of racial subgroups (White vs Black), PPB (*P* = .008), and primary treatment received (*P* = .01).

**Table 1:** Cohort Characteristics (n = 442).

**Characteristics**	**ROI: no, n = 96**	**ROI: yes, n = 346**	***P*****value**^a^
Age (years)			
Median (IQR)	69.5 (65, 74.5)	69 (62, 73)	
Age at diagnosis (n [%])			.5
< 65	31 (32.29 %)	131 (37.86 %)	
≥ 65	65 (67.71 %)	215 (62.14 %)	
Race (n [%])			.03*
White	73 (76.04 %)	302 (87.28 %)	
Black	13 (13.54 %)	24 (6.94 %)	
Other	10 (10.42 %)	20 (5.78 %)	
Prebiopsy PSA, ng/mL			.07
Median (IQR)	5.69 (4.27–8.26)	5.30 (3.90–7.50)	
Biopsy Gleason score, No. (%)			.001
NT	6 (6.25 %)	89 (25.72 %)	
GG1	30 (31.25 %)	96 (27.75 %)	
GG2	40 (41.67 %)	113 (32.66 %)	
GG3	14 (14.58 %)	25 (7.23 %)	
GG4	2 (2.08 %)	10 (2.89 %)	
GG5	4 (4.17 %)	13 (3.76 %)	
PPB			.008
Median (IQR)	16.66 (8.33–40)	14.28 (8.33–25)	
Primary treatment			.01
NA	2 (2.08 %)	7 (2.02 %)	
No treatment	33 (34.38 %)	183 (52.89 %)	
Radical prostatectomy	16 (16.67 %)	43 (12.43 %)	
Radiation therapy	45 (46.88 %)	113 (32.66 %)	

Abbreviations: GG, Gleason grade group; IQR, interquartile range; NA, not available; NT, no tumor; PPB, percent-positive biopsy cores; PSA, prostate-specific antigen; ROI, region of interest.

* *P* value taken for race category only takes White and Black into consideration.

a Wilcoxon rank-sum test for numeric and Pearson’s Chi-square test for categorical variables.

**Fig. 2:**
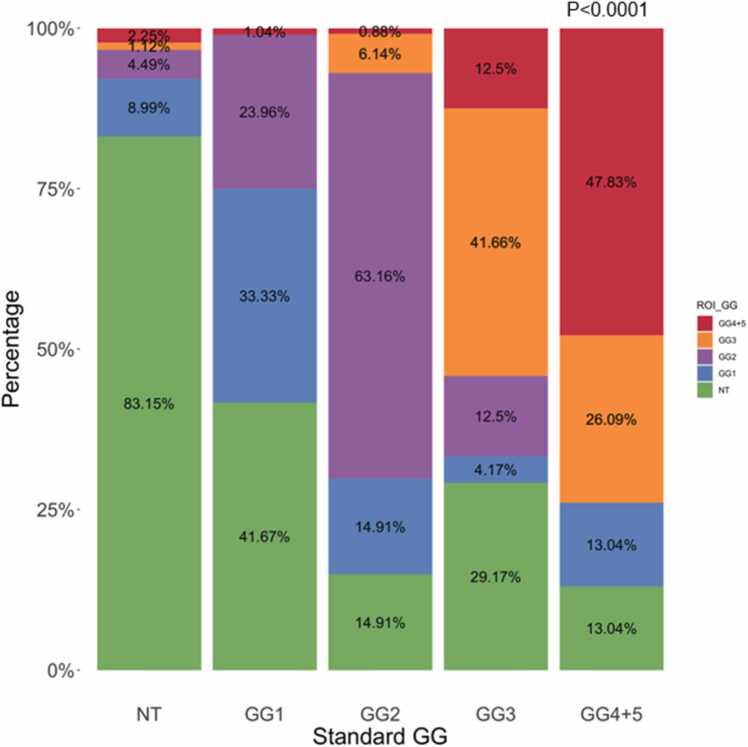
Gleason reclassification. Comparison of Gleason grade groups between standard biopsy and ROI biopsy. Abbreviations: GG, Gleason grade group; NT, no tumor; ROI, region of interest.

We focused on patients who underwent both standard and ROI biopsy (Supplementary Table 1 for Fig. 2). Among the men in cohort A who received a diagnosis of *no tumor* after standard biopsy, 16.9 % (n = 15) showed disease on subsequent ROI biopsy (Fig. 2), with around 8.0 % (n = 7) receiving a Gleason score of 7–10 (Fig. 2), indicating clinically significant cancer, which may have escaped detection on the original standard biopsy. Similarly, of all patients who had a Gleason grade group 1 (clinically nonsignificant cancer) tumor detected after standard biopsy, 25 % (n = 24) of them showed evidence of clinically significant high-risk cancer (grade group 2 and higher) after ROI biopsy (Fig. 2).

**Fig. 3:**
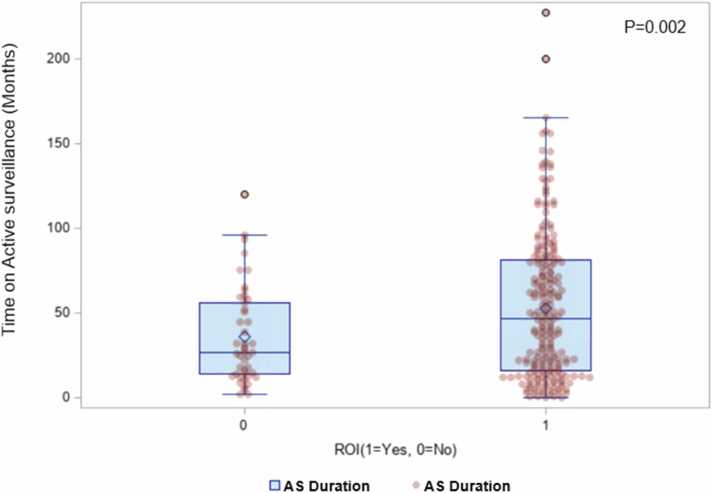
Comparison of time spent on active surveillance between both cohorts. Abbreviations: AS, active surveillance; ROI, region of interest.

Among those patients who had high-risk cancer detected after ROI biopsy, we investigated the impact of Gleason grade reclassification on treatment status using a multivariable logistic regression model, adjusting for prediagnostic variables (age at diagnosis, self-reported race, prebiopsy PSA). The results showed that reclassification affected treatment decision-making significantly (odds ratio = 2.91, CI = 1.30–6.51, *P* = .008) (Table 2). Reclassified patients were almost 3 times more likely to receive treatment compared with those whose Gleason scores were not changed (Table 2). VIF values for all confounders were around 1.0 (reclassification = 1.02, age at diagnosis = 1.02, prebiopsy PSA = 1.03, self-reported race = 1.0), indicating no multicollinearity between these variables (Table 2). Model calibration using Hosmer-Lemeshow test yielded a P-value of 0.74, suggesting a great fit of the model to the data.

**Table 2:** Impact of Reclassification and Prediagnostic Variables on Treatment Status.

**Variables**	Odds ratio	95 % CI	*P* value	**VIF**	**AUC**
Reclassification					
Yes vs no	2.91	1.30–6.51	0.008	1.02	
Age	1.03	0.98–1.08	0.2	1.02	0.63
Prebiopsy PSA	1.01	0.91–1.12	0.7	1.03	
Race					
Black vs White	2.11	0.58–7.65	0.2	1.00	

Abbreviations: AUC, area under curve; PSA, prostate-specific antigen.

Multivariable model is adjusted for Gleason reclassification, age at diagnosis, Prebiopsy PSA, and self-identified race. Model calculates the predictive odds of undergoing treatment.

**Fig. 4:**
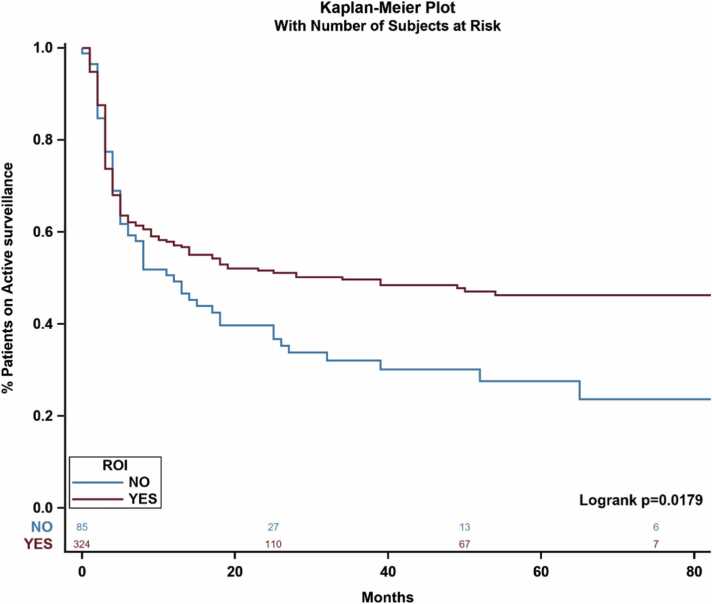
Impact of ROI biopsies on time-to-first treatment and likelihood of receiving primary treatment. Abbreviations: ROI, region of interest.

**Fig. 5:**
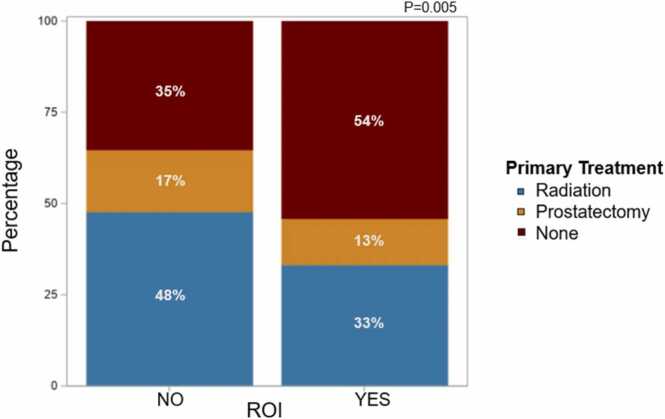
Comparison between different treatment types within both cohorts. Abbreviations: AS, active surveillance; ROI, region of interest.

In terms of time-to-treatment analysis, ROI biopsy was associated significantly with remaining on AS and delaying the primary treatment. Patients who received ROI biopsy in addition to standard TRUS biopsy were less likely to receive primary treatment, while 54 % remained on AS (Fig. 5). The time to receive primary treatment was significantly higher in the ROI group compared with the non-ROI group (*P* = .017) (Fig. 4). Furthermore, among patients who underwent AS, the median time spent on AS was significantly longer for the ROI group compared with the non-ROI cohort (*P* = .002) (Fig. 3).

In terms of Gleason reclassification among the AS group within cohort A, ROI biopsy demonstrated high accuracy in predicting increase in Gleason grade. Among the men in cohort A who received a diagnosis of *no tumor* after standard biopsy and were recommended AS, 15 % (n = 11) showed disease on subsequent ROI biopsy (Supplementary Figure 1), with around 4.0 % (n = 3) receiving a Gleason score of 7–10 (Supplementary Figure 1), indicating clinically significant cancer, which may have escaped detection on the original standard biopsy. Similarly, of all patients who had a Gleason grade group 1 (clinically nonsignificant cancer) tumor detected after standard biopsy and were recommended AS, 22 % (n = 15) of them showed evidence of clinically significant high-risk cancer (grade group 2 and higher) after ROI biopsy (Supplementary Figure 1).

**Fig. 6:**
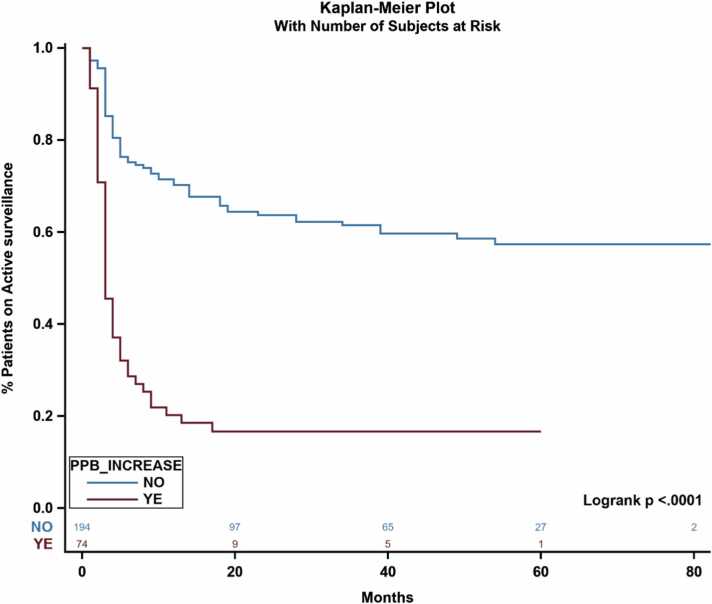
Impact of PPB increase on time-to-first treatment and likelihood of receiving primary treatment. Abbreviations: MRI, magnetic resonance imaging; PPB, percent-positive biopsy core.

MRI fusion biopsies were specifically targeted at areas of suspicion identified on the MRI, leading to a greater likelihood of sampling cancerous tissue. When comparing changes in PPB between MRI fusion biopsy and standard biopsy in cohort A, PPB increase was significantly associated with lower time spent on AS (*P* < .001) (Fig. 6). More than 50 % of patients who did not have an increase in PPB after MRI biopsy remained on AS without undergoing any definitive treatment (Fig. 6).

When investigating the effect of prediagnostic variables (age at diagnosis, race, prebiopsy PSA, and ROI) on time-to-treatment, ROI status (aHR = 0.71, CI = 0.52–0.97, *P* = .03), race (White vs Black) (aHR = 1.60, CI = 1.03–2.49, *P* = .03), and prebiopsy PSA (aHR = 1.04, CI = 1.01–1.06, *P* = .0008) all were significantly associated with time-to-treatment (Table 3).

**Table 3:** Cox Proportional HR Using Prediagnostic Variables.

	**Unadjusted**			**Adjusted**		
**Variable**	**HR**	**95 % CI**	***P*****value**	**HR**	**95 % CI**	***P*****value**
ROI status						
No	1	—	—	1	—	—
Yes	0.7	0.51–0.95	.02	0.71	0.52–0.97	.03
Age at diagnosis						
< 65	1	—	—	1	—	—
≥ 65	1.09	0.82–1.46	.4	1.08	0.83–1.41	.5
Race						
White	1	—	—	1	—	—
Black	1.63	1.06–2.50	.02	1.60	1.03–2.49	.03
Other	0.97	0.55–1.72	.9	0.95	0.53–1.68	.8
Prebiopsy PSA	1.04	1.01–1.06	.0004	1.04	1.01–1.06	.0008

Abbreviations: HR, hazard ratio; PSA, prostate-specific antigen; ROI, region of interest.

Multivariable model is adjusted for ROI status, age at diagnosis, Prebiopsy PSA, and self-identified race. Model investigates the effects of prediagnostic variables on time-to-treatment.

When ROI status, age, and race were compared as 3 prediagnostic variables, both ROI status (ROI [yes]: aHR = 0.71, CI = 0.52–0.97, *P* = .03); (ROI [no]: aHR = 1.39, CI = 1.02–1.90, *P* = .03) and race (White vs Black) (aHR = 1.63, CI = 1.05–2.54, *P* = .02) were significantly associated with time-to-treatment (Supplementary Tables 2, 3 for Table 3).

## 4 Discussion

Both mpMRI and TRUS biopsies have previously been proven to be clinically valid tests for detecting PCa. [15] Although both biopsies are useful modes of detection, their approaches are vastly different and some discrepancy between the two is expected. [2], [5], [7], [16] Within cohort A, there are clear discrepancies between the Gleason scores and cancer grading obtained from standard biopsy vs targeted ROI mpMRI biopsy; this suggests a clinical utility in combining the two techniques for certain demographics. In the cohort of patients whose cancer grade on TRUS biopsy was grade 2 or higher, the discrepancy with mpMRI biopsy can largely be ignored clinically, because these patients will often be recommended for radical treatment regardless. [17] Thus, we posit that the ROI biopsy may have the most clinical utility for diagnosing and detecting PCa among individuals who were originally thought to have no tumor or only grade 1 cancer on TRUS biopsy.

SEER data suggests that PCa is a remarkably treatable disease with a 97.1 % 5-year overall survival. [1] Further dissection of the data, however, reveals that while the 5-year survival of localized PCa approaches 100 %, the 5-year expected survival of patients with distant or metastasized PCa is just 34.1 %. [1] This prominent drop in survival highlights the potential role of mpMRI biopsy among patients originally classified as *no tumor* or grade group 1 with standard TRUS biopsy. Our data suggests that 7.9 % of patients who were considered as *no tumor* and 25 % of patients who were categorized as grade group 1 may be reclassified to grade group 2 or higher with mpMRI biopsy. Our findings are consistent with recently published similar studies, which showed 20 %-25 % increase in detection of csPCa using mpMRI biopsy [18], [19]. Furthermore, among patients who were recommended AS, mpMRI biopsy detected increase in Gleason grade among 4.11 % of patients who were considered as *no tumor* and 22 % of patients who were categorized as grade group 1 which would have clear implications for physician decision-making regarding radical treatment vs AS. Additionally, an increase in PPB associated with mpMRI biopsy was significantly associated with a corresponding decrease in time spent on AS. Although discrepancies between mpMRI and standard TRUS biopsy persisted in the higher-grade groups, these may not be clinically relevant; regardless of the mpMRI biopsy findings, patients with grade group 5 disease, for example, are known to have clinically significant PCa would not be offered AS. [17] Therefore, we suggest that the true clinical utility of adding the mpMRI biopsy lies in using this second form of biopsy as a confirmatory tool for patients with no tumor or grade group 1 disease, for whom reclassification may have drastic treatment implications.

Conversely, ROI biopsy results which confirm the existence of no tumor or low-grade cancer (Gleason score of 6) could be clinically useful in validating the standard biopsy results, making both clinicians and patients more confident in recommending AS or no action. This increased confidence of patients and providers in AS could help in combating the overtreatment of low-grade PCa and the subsequent deleterious and unnecessary side effects. Radical prostatectomy has a myriad of established side effects, which can drastically and negatively impact the quality of patients’ lives because of erectile dysfunction, [20] urinary incontinence, [20], [21] and inguinal hernia. [22], [23] Previous literature strongly suggests that patients with localized PCa experience a better quality of life if they are placed on AS opposed to immediately undergoing radical prostatectomy; the impact of this treatment is seen across several domains, including both sexual and urinary function. [24], [25], [26] Thus, using mpMRI biopsy as a confirmatory test to increase both patient and provider satisfaction with AS may play an important role in preserving quality of life.

This observation is supported by the results of the time-to-treatment analysis. Individuals in cohort A who received ROI biopsy had a significant increase in time to receive primary treatment compared with individuals who received only standard biopsy. These results could be produced by multiple factors, including a delay enforced by needing a secondary procedure (the ROI biopsy itself), or increased confidence in diagnosing low-grade PCa, which would not necessitate immediate treatment. The increase in time to receive primary treatment suggests that using mpMRI biopsy as an adjunct to standard TRUS biopsy has a potential impact on clinical decision-making. ROI biopsy, therefore, may present as a valuable tool for clinicians to combat the overtreatment of patients whose PCa would not benefit from radical treatment, patients who would suffer the consequences of unnecessary radiation therapy or prostatectomy. Previous literature has suggested that adding an ROI biopsy can increase detection of clinically significant PCa [5] while simultaneously decreasing detection of clinically insignificant PCa [4], [7] combating overdiagnosis.

Initial overdiagnosis of PCa drives overtreatment, with potentially profound consequences for patients. A systematic review by Fenton et al. [27] for the United States Preventive Services Task Force found that up to 50.4 % of screen-detected prostate cancers may be associated with overdiagnosis, indicating that these cancers were unlikely to become clinically significant during the patient’s lifetime [27]. [28] These findings influenced the Task Force’s current recommendation that the decision to undertake PCa screening for patients between 55 and 69 years of age should be an individualized one, as there is currently not enough evidence to firmly suggest that the benefits of treatment outweigh the potential risk of overtreatment. [29] Evidence suggests treatment, such as radical prostatectomy, can have significant consequences for the patient and is associated with an increased risk of urinary incontinence and erectile dysfunction when compared with conservative management. [28] Using ROI biopsy and its associated increase in time to receive primary treatment can, therefore, help in maintaining optimal genitourinary function for patients.

Considering that this is a single-center clinical study, results from this study should be cautiously applied to the general population. Furthermore, the relationship between receiving an mpMRI biopsy and time-to-treatment previously explored is descriptive in nature, and we are aware of this limitation and are not seeking to establish a causal relationship between the two. Another potential limitation of MRI fusion biopsy is the high cost associated with the procedure, which may limit accessibility and widespread adoption, particularly among resource-limited settings. However, utilizing data from the Centers for Medicare and Medicaid Services from 2013 regarding the processing of prostate biopsy specimens, it's estimated that adopting a fusion biopsy-only approach could lead to cost savings that would cover 97 % of the expenses associated with prostate MP-MRI in comparison to the conventional 12-core biopsy, due to the reduction in the number of individual biopsy specimens submitted (which are reimbursed separately). Additionally, variability in MRI interpretation can impact the accuracy of lesion targeting, leading to inconsistency among identification of csPCa. Lastly, during the study timespan (2011–2018), there have been changes with the PIRADs score of MRI, and a different scoring system could impact biopsy results. Despite these limitations, the results from this work may offer the field new insight into the future clinical directions of the utility of mpMRI biopsy in detecting PCa.

## 5 Conclusion

Adding ROI biopsy to standard PCa screenings represents a valuable opportunity to increase detection of clinically significant cancer among patients originally classed as *no tumor* or grade group 1 on standard biopsy. This increased detection has serious treatment implications, as patients with grade group 2 disease or above would often benefit from beginning immediate treatment. Furthermore, our findings suggest that using mpMRI biopsy as a confirmatory test to increase both patient and provider satisfaction with AS may play an important role in preserving the quality of life by decreasing the risk of overtreatment and reducing treatment-related costs.

## Ethics and consent statement

The study was approved by the institutional review board of Advarra (#00000971), Columbia, Maryland. The institutional review board number is Pro00015023. Current date of approval is from June 7, 2024, to June 7, 2025. Analyses were conducted with an approved waiver for obtaining informed consent and with authorization by the Health Insurance Portability and Accountability Act of 1996.

## Funding information

Cancer Center Support grant P30-CA076292 to 10.13039/100009164Moffitt Cancer Center.

## CRediT authorship contribution statement

**Katende Esther:** Writing – review & editing, Project administration, Methodology, Data curation, Conceptualization. **Trivedi Purvish:** Writing – original draft, Visualization, Validation, Software, Methodology, Formal analysis, Data curation, Conceptualization. **Smith Riley:** Resources, Methodology, Data curation. **Awasthi Varun:** Writing – original draft, Methodology, Formal analysis, Data curation. **Yamoah Kosj:** Writing – review & editing, Validation, Supervision, Resources, Methodology, Investigation, Funding acquisition, Conceptualization. **Dhillon Jasreman:** Writing – review & editing, Validation, Supervision, Conceptualization. **Wang Carrie:** Writing – review & editing, Writing – original draft, Methodology, Conceptualization. **Park Jong Y.:** Writing – review & editing, Validation, Supervision, Methodology, Conceptualization. **Bondokji Yahya:** Validation, Methodology, Data curation. **Putney Ryan:** Visualization, Software, Methodology, Formal analysis.

## Declaration of Competing Interest

The authors declare no potential conflicts of interest.

## Data Availability

The data that support the findings of this study are available from the corresponding author KY upon reasonable request. The data are not publicly available due to privacy concerns.

## References

[1] Cancer of the Prostate Accessed 09/05/2023. 〈https://seer.cancer.gov/statfacts/html/prost.html〉.

[2] Kasivisvanathan V., Rannikko A.S., Borghi M. (May 10 2018). MRI-targeted or standard biopsy for prostate-cancer diagnosis. N. Engl. J. Med.

[3] Wegelin O., Exterkate L., van der Leest M. (Apr 2019). The future trial: a multicenter randomised controlled trial on target biopsy techniques based on magnetic resonance imaging in the diagnosis of prostate cancer in patients with prior negative biopsies. Eur. Urol..

[4] van der Leest M., Cornel E., Israël B. (Apr 2019). Head-to-head comparison of transrectal ultrasound-guided prostate biopsy versus multiparametric prostate resonance imaging with subsequent magnetic resonance-guided biopsy in biopsy-naïve men with elevated prostate-specific antigen: a large prospective multicenter clinical study. Eur. Urol..

[5] Aminsharifi A., Gupta R.T., Tsivian E., Sekar S., Sze C., Polascik T.J. (Jan 2019). Reduced Core Targeted (RCT) biopsy: Combining multiparametric magnetic resonance imaging - transrectal ultrasound fusion targeted biopsy with laterally-directed sextant biopsies - an alternative template for prostate fusion biopsy. Eur. J. Radio..

[6] Giannakodimos I., Kaltsas A., Moulavasilis N. (Jan 12 2025). Fusion MRI/ultrasound-guided transperineal biopsy: a game changer in prostate cancer diagnosis. J. Clin. Med.

[7] Siddiqui M.M., Rais-Bahrami S., Turkbey B. (Jan 27 2015). Comparison of MR/ultrasound fusion-guided biopsy with ultrasound-guided biopsy for the diagnosis of prostate cancer. Jama.

[8] Ahmed H.U., El-Shater Bosaily A., Brown L.C. (2017). Diagnostic accuracy of multi-parametric MRI and TRUS biopsy in prostate cancer (PROMIS): a paired validating confirmatory study. Lancet.

[9] Lu Y.T., Delijani K., Mecum A., Goldkorn A. (2019). Current status of liquid biopsies for the detection and management of prostate cancer. Cancer Manag Res.

[10] Lomas D.J., Ahmed H.U. (Jun 2020). All change in the prostate cancer diagnostic pathway. Nat. Rev. Clin. Oncol..

[11] von Elm E., Altman D.G., Egger M. (Dec 2014). The strengthening the reporting of observational studies in epidemiology (STROBE) statement: guidelines for reporting observational studies. Int J. Surg..

[12] van Leenders G., van der Kwast T.H., Grignon D.J. (Aug 2020). The 2019 international society of urological pathology (ISUP) consensus conference on grading of prostatic carcinoma. Am. J. Surg. Pathol..

[13] Wei J.T., Barocas D., Carlsson S. (Jul 2023). Early detection of prostate cancer: AUA/SUO guideline part i: prostate cancer screening. J. Urol..

[14] Wei J.T., Barocas D., Carlsson S. (Jul 2023). Early detection of prostate cancer: AUA/SUO guideline part ii: considerations for a prostate biopsy. J. Urol..

[15] Rouvière O., Puech P., Renard-Penna R. (Jan 2019). Use of prostate systematic and targeted biopsy on the basis of multiparametric MRI in biopsy-naive patients (MRI-FIRST): a prospective, multicentre, paired diagnostic study. Lancet Oncol..

[16] De Luca S., Fiori C., Bollito E. (Jun 2020). Risk of Gleason Score 3+ 4= 7 prostate cancer upgrading at radical prostatectomy is significantly reduced by targeted versus standard biopsy. Minerva Urol. Nefrol..

[17] Mohler J.L., Antonarakis E.S., Armstrong A.J. (May 1 2019). Prostate cancer, version 2.2019, NCCN Clinical Practice Guidelines in Oncology. J. Natl. Compr. Canc Netw..

[18] Rastinehad A.R., Turkbey B., Salami S.S. (Jun 2014). Improving detection of clinically significant prostate cancer: magnetic resonance imaging/transrectal ultrasound fusion guided prostate biopsy. J. Urol..

[19] Ahdoot M., Lebastchi A.H., Gharam S. (2019). MRI targeted biopsy dramatically increases detection of clinically significant prostate cancer while reducing the risk of indolent cancer detection. J. Clin. Oncol..

[20] Clavell-Hernández J., Martin C., Wang R. (Jan 2018). Orgasmic dysfunction following radical prostatectomy: review of current literature. Sex. Med Rev..

[21] Frey A.U., Sønksen J., Fode M. (Feb 2014). Neglected side effects after radical prostatectomy: a systematic review. J. Sex. Med.

[22] Zhu S., Zhang H., Xie L., Chen J., Niu Y. (Mar 2013). Risk factors and prevention of inguinal hernia after radical prostatectomy: a systematic review and meta-analysis. J. Urol..

[23] Ichioka K., Yoshimura K., Utsunomiya N. (Feb 2004). High incidence of inguinal hernia after radical retropubic prostatectomy. Urology.

[24] Lardas M., Liew M., van den Bergh R.C. (Dec 2017). Quality of life outcomes after primary treatment for clinically localised prostate cancer: a systematic review. Eur. Urol..

[25] van den Bergh R.C., de Blok W., van Muilekom E., Tillier C., Venderbos L.D., van der Poel H.G. (Aug 2014). Impact on quality of life of radical prostatectomy after initial active surveillance: more to lose?. Scand. J. Urol..

[26] Jeldres C., Cullen J., Hurwitz L.M. (Jul 15 2015). Prospective quality-of-life outcomes for low-risk prostate cancer: Active surveillance versus radical prostatectomy. Cancer.

[27] Fenton J.J., Weyrich M.S., Durbin S., Liu Y., Bang H., Melnikow J. (2018). Prostate-Specific Antigen–Based Screening for Prostate Cancer: Evidence Report and Systematic Review for the US Preventive Services Task Force. JAMA.

[28] Fenton J.J., Weyrich M.S., Durbin S., Liu Y., Bang H., Melnikow J. (May 8 2018). Prostate-specific antigen-based screening for prostate cancer: evidence report and systematic review for the US preventive services task force. Jama.

[29] Grossman D.C., Curry S.J., Owens D.K. (May 8 2018). Screening for prostate cancer: US preventive services task force recommendation statement. Jama.

