# Supplementary material for: Role of region-of-interest magnetic resonance imaging fusion biopsy in mitigating overtreatment of localized prostate cancer – A retrospective cohort study
Source: Eur J Radiol Open. 2025 Mar 7;14:100642. doi: 10.1016/j.ejro.2025.100642 (PMC11930199; doi:10.1016/j.ejro.2025.100642)
Supplement: Supplementary file 1 — Supplementary material [file mmc1.docx]

**Supplemental Table 1: Focus group for Gleason reclassification**

| **Standard GS Grade Group** | **Total Participants** | **ROI Biopsy: Yes** |
| --- | --- | --- |
| No Tumor (NT) | 94 | 89 |
| Grade Group 1 (3+3) | 115 | 96 |

**Abbreviations:** Standard GS Grade Group, Standard Gleason Grade group; ROI, region of interest.

Focus group for Gleason reclassification. Patients who underwent both Standard and ROI biopsy (n = 185).

**Supplemental Table 2: Cox-Proportional Hazard ratio using prediagnostic variables**

|  | **Unadjusted** | | | **Adjusted** | | |
| --- | --- | --- | --- | --- | --- | --- |
| **Variable** | **HR** | **95% CI** | **P value** | **HR** | **95% CI** | **P value** |
| **ROI Status** |  |  |  |  |  |  |
| No | 1 | - | - | 1 | - | - |
| Yes | 0.7 | 0.51-0.95 | .02 | 0.71 | 0.52-0.97 | .03 |
| **Age at diagnosis** |  |  |  |  |  |  |
| <65 | 1 | - | - | 1 | - | - |
| >=65 | 1.09 | 0.82-1.46 | .4 | 1.09 | 0.84-1.41 | .4 |
| **Race** |  |  |  |  |  |  |
| White | 1 | - | - | 1 | - | - |
| Black | 1.63 | 1.06-2.50 | .02 | 1.63 | 1.05-2.54 | .02 |
| Other | 0.97 | 0.55-1.72 | .9 | 0.96 | 0.54-1.70 | .8 |

**Abbreviations:** CI, Confidence Interval; HR, hazard ratio; ROI, region of interest.

Multivariable model to evaluate the effects of prediagnostic variables on time-to-treatment.

**Supplemental Table 3: Cox-Proportional Hazard ratio using prediagnostic variables**

|  | **Unadjusted** | | | **Adjusted** | | |
| --- | --- | --- | --- | --- | --- | --- |
| **Variable** | **HR** | **95% CI** | **P value** | **HR** | **95% CI** | **P value** |
| **ROI Status** |  |  |  |  |  |  |
| Yes | 1 | - | - | 1 | - | - |
| No | 1.42 | 1.05-1.94 | .02 | 1.39 | 1.02-1.90 | .03 |
| **Age at diagnosis** |  |  |  |  |  |  |
| <65 | 1 | - | - | 1 | - | - |
| >=65 | 1.09 | 0.82-1.46 | .4 | 1.09 | 0.84-1.41 | .4 |
| **Race** |  |  |  |  |  |  |
| White | 1 | - | - | 1 | - | - |
| Black | 1.63 | 1.06-2.50 | .02 | 1.63 | 1.05-2.54 | .02 |
| Other | 0.97 | 0.55-1.72 | .9 | 0.96 | 0.54-1.70 | .8 |

**Abbreviations:** CI, Confidence Interval; HR, hazard ratio; ROI, region of interest.

Multivariable model to evaluate the effects of prediagnostic variables on time-to-treatment.

**Supplemental figure 1: Gleason reclassification among Active surveillance group within Cohort A**

**
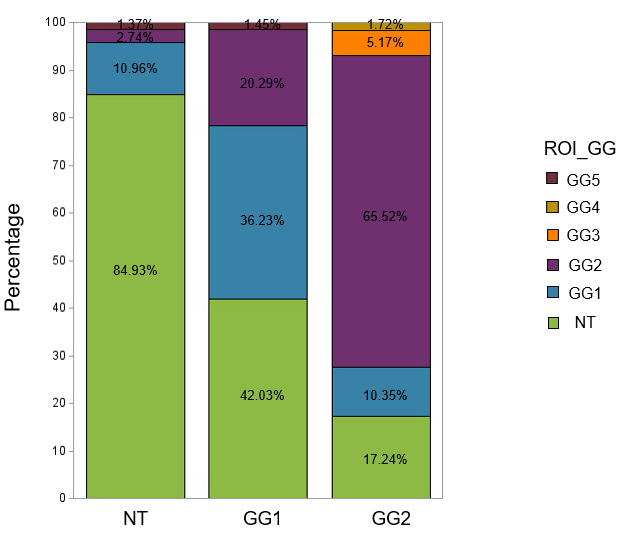
**

**Abbreviations:** ROI, region of interest; GG, Gleason grade group; NT, no tumor.

Gleason reclassification. Comparison between standard biopsy and ROI biopsy Gleason grade groups among patients undergoing active surveillance.
